# A Very Rare Presentation of Open Dislocation of the Elbow in a Child: A Case of Total Section of Brachial Artery and Present Distal Pulse with Literature Review

**DOI:** 10.1055/s-0043-1770968

**Published:** 2024-12-27

**Authors:** Cherrabi Hind, Abdelkarim Kharroubib, Mohamed Amine Oukhouyab, Fatima Smahia, Badr Elkassimi

**Affiliations:** 1 Departamento de Cirurgia Pediátrica, Faculdade de Medicina e Farmácia de Agadir, Ibn Zohr University, Agadir, Marrocos; 2 Departamento de Cirurgia Vascular, Faculdade de Medicina e Farmácia de Agadir, Ibn Zohr University, Agadir, Marrocos

**Keywords:** brachial artery, child, elbow, range of motion, articular, pulse

## Abstract

Open elbow dislocation is a rare emergency in children. Its association with brachial artery injury is very rare. We report the case of a child admitted for total section of the brachial artery on open dislocation of the elbow before absence of signs of ischemia of the upper limb concerned as well as a review of the literature which is very poor.

## Introduction

Dislocations of the elbow joint in children are not common. 1 Of all elbow injuries in skeletally immature patients, Henrikson found that only ∼3% of all were dislocations. 2

Lesions of the brachial artery are more commonly described in supra-condylar fractures in children, isolated or accompanying a nerve injury.

Nevertheless, there is very little data in the literature regarding open elbow dislocation in children and its presentation associated with total section of the brachial artery is very rare. We present such a rare case of a child admitted to the pediatric emergency room for open dislocation of the elbow, the vascular examination revealed radial and ulnar pulses present in whom the surgical exploration revealed the total section of the brachial artery.

## Case Report

This is a 9-year-old boy without any particular pathological history who fell from an estimated height of 4m, within 12 hours before his admission, with a landing on the right upper limb causing a deformation of the right elbow associated with a total functional impotence of the right upper limb and an anterior wound of the right elbow and a hematoma opposite. The ulnar and radial pulses were present but weak; the warm limb had normal coloring in a hemodynamically stable child.

**Fig. 1:**
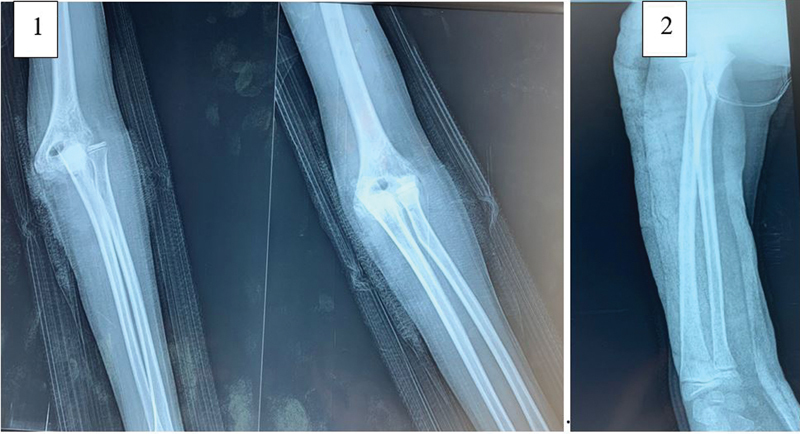
Standard preoperative radiograph showing posterolateral elbow dislocation.

**Fig. 2:**
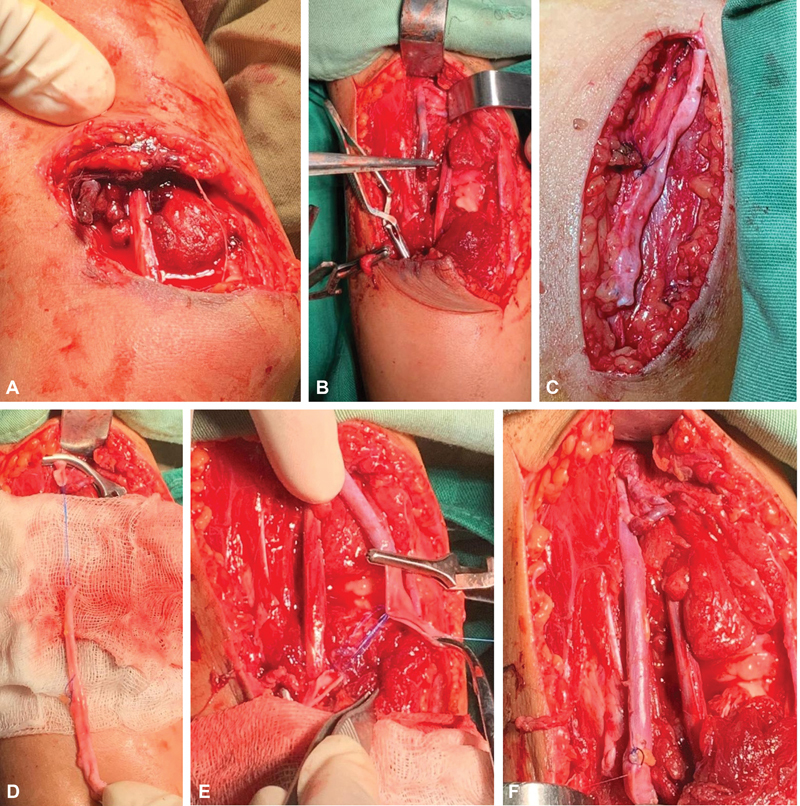
Standard radiograph showing reduction of the elbow dislocation. ( **A** ) Intraoperative image showing the anterior elbow wound with the presence of the hematoma; ( **B** ) demonstration of the two severed ends of the brachial artery; ( **C, D** ) preparation of the reverse saphenous vein graft of the right thigh; ( **E** ) presentation of the distal venous segment to be anastomosed with the distal end of the severed brachial artery; ( **F** ) final result after terminal anastomosis and verification of hemostasis.

The standard X-ray showed a posterolateral dislocation of the right elbow without any other associated fracture, especially of the epitrochlea ( Figs. 1 , 2 ).

The patient was admitted for surgical exploration: a huge hematoma was found in front of the medial part of the right elbow masking the total frank section of the brachial artery thrombosed on its proximal end associated with a contusion of the median nerve.

After reduction of the posterolateral dislocation of the elbow and verification of its stability, the brachial artery was repaired by placement of a reverse saphenous vein graft sutured with 6/0 poly-prolene in two hemi-surjects at each end. The patient's right upper limb was immobilized postoperatively in a brachio-palmar splint in flexion (intraoperative images a, b, c, d, e, f).

## Discussion

Elbow dislocation in children is less common than elbow fractures, especially of the supra condyle, 3 lateral condyle, epitrochlea, upper radial and ulnar extremities.

There are very few data in the literature addressing post-traumatic brachial artery injury in elbow dislocation in the pediatric population compared with the series described in adults.

The posterior variant of the elbow dislocation is by far the most frequent secondary to the transmission of force from an indirect impact on the hand in extension during the fall.

In ∼60% of cases, it is posterolateral, rarely divergent or anterior due to the direct impact on the elbow and exceptionally convergent. 4

In first-line supracondylar fractures, the authors state that the ulnar nerve should be explored during surgery. Whether or not the median radial nerve and blood vessel should be explored should be decided by the results of the preoperative examination. 5

Open reduction and exploration of the injured nerve is not necessarily indicated for nerve injury in a closed fracture. Neural recovery, regardless of the injured nerve, usually occurs under observation but may take 6 months or more. 1

Elbow dislocation may be isolated or associated with other fractures commonly the epicondyle fracture (defining Watson-Jones type 4), the radial superior extremity, the coronoid process and less frequently the lateral condyle, the olecranon, the capitulum and the trochlea. This lesion association was reported in 63% of cases in the series by Carlioz and Abols, 5 in 54% of cases in the series by Rasool. 6

Damage to one or more elements of the vascular-nervous pedicle accompanies elbow dislocation in children when it is open and less frequently in closed dislocation. 7

The dislocation of the elbow may, depending on the direction of displacement, interfere with the path of the nerve elements, in this case: the ulnar nerve medially, the median nerve anteriorly and the radial nerve posteriorly and externally.

Some authors have reported the case of an elbow dislocation in a child by entrapment of the median nerve and paralysis of the ulnar nerve after reduction. 8 this underlines the necessity of a meticulous and comparative neurological examination evaluating the sensitive and motor function of each nerve and oriented by the direction of the displacement objectified by the radiological assessment

The brachial artery in its course is found next to the medial edge of the biceps brachii muscle before becoming, together with the median nerve, more superficial and covered by the brachial aponeurosis 9 which explains its exposure during fracture displacements.

The richness of the collateral of vascularization concerning the elbow explains the conservation of blood flow, the rarity of the installation of ischemia as well as the vascular suffering of the upper limb reached, mainly the decrease of distal pulsations, cold, paleness, cyanosis of the limb and, to a lesser degree, edema. 10

Most often, especially in closed elbow dislocations, the decrease in radial and/or ulnar pulses is secondary to the compression of the humeral axis by the displacement of the lower end of the humerus and the pulse is recovered after the dislocation is reduced.

Nevertheless, when there is a doubt about the presence of distal pulses or the local condition of the upper limb, repeated close clinical examinations are necessary after the urgent reduction of the dislocation. The authors refer to the use of pulse oximetry, which is really a readily available tool, but how this is done needs to be clarified, i.e., looking at the wave form and not the oximetry complemented by angiography, preferably venous angiography, given the risk of arterial thrombosis. If angiography is not available, Doppler ultrasound can detect partial or total interruption of arterial flow. The slightest doubt in front of the persistence of the signs of ischemia the surgical exploration is indicated. This is the first recourse in open elbow dislocations.

The lesions of the brachial artery have several nuances, from simple contusion, spasm, partial section to thrombosis (by intimal flap) and total section. It is frequently described in children in supra condylar fractures and rarely in open dislocations and still exceptionally in closed dislocations. 11

This arterial damage may be associated with nerve damage due to the contiguity of the anatomical vascular-nerve relationships and depending on the direction of the displacement and its severity ( Table 1 ). 5 6 9 10 11 12 13

**Table 1:** Literature review of the vascular-nerve injury association in pediatric elbow dislocation

Author	Number of cases (children)	Complications	Results (%) Good and excellent
Carlioz, Abols 5	58	Ulnar nerve injury 2 cases	90%
Rassol 6	33	Ulnar nerve injury 2 cases Median nerve injury 1 case Radial nerve injury 1 case **Brachial artery injury 1 case**	67%
Subasi et al. 12	56	Ulnar nerve injury 3 cases **Median nerve injury and brachial artery injury 1 case**	48%
Louis et al. 13	1	**Rupture of the brachial artery 1 case**	Not reported
Hoffmann et al. 9	1	**Rupture of the brachial artery 1 case**	Not reported
Manouel et al. 10	1	**Rupture of the brachial artery 1 case**	Not reported
Brahmamdam et al. 11	1	**Rupture of the brachial artery 1 case**	Not reported

The repair of the brachial artery section was considered in the multi-disciplinary approach of the pediatric surgeon, the vascular surgeon and the anesthesia-intensive care team.

The upper limb as well as the lower limb were prepared;the latter being for a possible venous graft.

The inventory of the injury assessment revealed: the total rupture of the brachial artery in the presence of a large hematoma that was evacuated. The contusion of the median nerve was noted, which remains continuous. The ulnar and radial nerves were intact without obvious tendon or ligament damage.

The ulnar and radial nerves were intact with no obvious tendon or ligament damage. We proceeded to reduce the posterior dislocation of the elbow, which seemed stable after reduction.

The use of a reverse saphenous vein graft from the right thigh proved to be essential given the tension of the anastomosis. at the end of the operation, a plaster cast immobilization was performed.

Elbow dislocation associated with brachial artery injury is a very rare trauma emergency in the pediatric population.

The repetitive clinical examination is crucial, complemented by arterial Doppler ultrasound and/or angiography, without delaying surgical exploration at the slightest doubt.
