# Supplementary material for: A Very Rare Presentation of Open Dislocation of the Elbow in a Child: A Case of Total Section of Brachial Artery and Present Distal Pulse with Literature Review
Source: Rev Bras Ortop (Sao Paulo). 2024 Dec 27;59(Suppl 2):e238–42. doi: 10.1055/s-0043-1770968 (PMC11679625; doi:10.1055/s-0043-1770968)
Supplement: Related texts

# Research article: Apresentação muito rara de luxação aberta do cotovelo em criança: Um caso de secção total da artéria braquial e presença de pulso distal com revisão de literatura

**Keywords:** amplitude de movimento articular, artéria braquial, cotovelo, criança, pulso arterial

## Abstract

A luxação aberta do cotovelo é uma emergência rara em crianças. Sua associação à lesão da artéria braquial é ainda mais rara. Relatamos o caso de uma criança atendida por secção total da artéria braquial decorrente de uma luxação aberta do cotovelo antes da ausência de sinais de isquemia do membro superior em questão. Também realizamos uma revisão da literatura, que é escassa.

## Introdução

As luxações da articulação do cotovelo são incomuns em crianças. 1 De todas as lesões do cotovelo em pacientes com esqueleto imaturo, Henrikson relatou que apenas cerca de 3% foram luxações. 2

Em crianças, as lesões da artéria braquial são mais comumente descritas em fraturas supracondilares, sendo isoladas ou acompanhadas por lesão neurológica.

No entanto, há poucos dados na literatura sobre a luxação aberta do cotovelo em crianças e sua associação à secção total da artéria braquial é muito rara. Apresentamos o caso raro de uma criança atendida no serviço de urgência pediátrica por luxação aberta do cotovelo cujo exame vascular indicou a presença de pulso radial e ulnar e a exploração cirúrgica revelou a secção total da artéria braquial.

## Relato de Caso

Um menino de 9 anos de idade, sem histórico médico relevante, caiu de uma altura estimada em 4 metros nas 12 horas anteriores à internação. O menino caiu sobre o membro superior direito, o que causou deformação do cotovelo direito associada a impotência funcional total do membro superior direito, uma ferida na porção anterior do cotovelo direito e um hematoma oposto. Os pulsos ulnar e radial estavam presentes, mas fracos; o membro era quente, tinha coloração normal e a criança era hemodinamicamente estável.

A radiografia simples mostrou a luxação posterolateral do cotovelo direito sem outra fratura associada, especialmente da epitróclea ( Figs. 1 e 2 ).

**Fig. 1:** Radiografia simples pré-operatória mostrando a luxação posterolateral do cotovelo.

**Fig. 2:** Radiografia simples pré-operatória mostrando a redução da luxação do cotovelo. ( **A** ) A imagem intraoperatória mostra a ferida no cotovelo direito e a presença de hematoma; ( **B** ) Demonstração das duas extremidades seccionadas da artéria braquial; ( **C, D** ) Preparo do enxerto reverso de veia safena na coxa direita; ( **E** ) Segmento venoso distal a ser anastomosado à extremidade distal da artéria braquial seccionada; ( **F** ) Resultado da anastomose terminal e verificação da hemostasia.

O paciente foi internado para exploração cirúrgica, em que foi constatado um grande hematoma anterior na parte medial do cotovelo direito. O hematoma mascarava a secção total franca da artéria braquial com trombose em sua extremidade proximal e associada à contusão do nervo mediano.

Depois da redução da luxação posterolateral do cotovelo e verificação de sua estabilidade, a artéria braquial foi submetida a reparo com a colocação de enxerto de veia safena reversa suturada com poliproleno 6/0 em duas hemissobreposições em cada extremidade. No período pós-operatório, o membro superior direito do paciente foi imobilizado com tala braquiopalmar em flexão (imagens intraoperatórias a, b, c, d, e e f).

## Discussão

Em crianças, a luxação do cotovelo é menos comum que as fraturas do cotovelo, especialmente das extremidades supracondilar, 3 condilar lateral, epitroclear, radial superior e ulnar.

Existem poucos dados na literatura sobre a lesão pós-traumática da artéria braquial por luxação do cotovelo na população pediátrica em comparação a adultos.

A variante posterior da luxação do cotovelo é de longe a mais frequente. É provocada pela transmissão de força de um impacto indireto na mão em extensão durante a queda.

Cerca de 60% dos casos são posterolaterais; as lesões raramente são divergentes ou anteriores devido ao impacto direto no cotovelo. Em casos excepcionais, são convergentes. 4

Nas fraturas supracondilares de primeira linha, o nervo ulnar deve ser explorado à cirurgia. A exploração ou não do nervo radial, do nervo mediano e dos vasos sanguíneos deve ser decidida de acordo com os achados do exame pré-operatório. 5

A redução aberta e a exploração do nervo lesionado não são necessariamente indicadas em lesões neurológicas no contexto de fraturas fechadas. A recuperação neurológica, independentemente do nervo acometido, de modo geral ocorre sob observação, mas pode levar 6 meses ou mais. 1

A luxação do cotovelo pode ser isolada ou associada a outras fraturas, em especial do epicôndilo (definindo o tipo 4 de Watson-Jones), da extremidade superior do rádio, do processo coronoide e, com menor frequência, do côndilo lateral, do olécrano, do capítulo umeral e da tróclea. Essa associação de lesões foi observada em 63% dos casos relatados por Carlioz e Abols 5 e em 54% dos casos descritos por Rasool. 6

Em crianças, a lesão de um ou mais elementos do pedículo neurovascular é vista em luxações abertas de cotovelo ou, com menor frequência, fechadas. 7

Dependendo de sua direção, a luxação do cotovelo pode interferir no trajeto dos elementos nervosos. Nestes casos, há acometimento do nervo ulnar medialmente, do nervo mediano anteriormente e o nervo radial posterior e externamente.

Alguns autores relataram o caso de uma luxação de cotovelo em uma criança com aprisionamento do nervo mediano e paralisia do nervo ulnar após a redução. 8 Isso reforça a necessidade de um exame neurológico meticuloso e comparativo, com avaliação da função sensorial e motora de cada nervo e orientado pela direção da luxação, que é revelada pela avaliação radiológica.

O trajeto da artéria braquial é próximo à borda medial do músculo bíceps braquial. Depois, a artéria braquial, juntamente com o nervo mediano, se torna mais superficial e é recoberta pela aponeurose braquial, 9 o que explica sua exposição em luxações e fraturas.

A riqueza de vascularização colateral no cotovelo esclarece a conservação do fluxo sanguíneo e a raridade da isquemia e do sofrimento vascular do membro superior acometido, principalmente em relação à diminuição dos pulsos distais, frio, palidez, cianose e, em menor grau, edema do membro. 10

De modo geral, principalmente nas luxações fechadas do cotovelo, a diminuição do pulso radial e/ou ulnar é secundária à compressão do eixo umeral pelo deslocamento da extremidade inferior do úmero; dessa forma, há recuperação do pulso após a redução da luxação.

Ainda assim, em caso de dúvida sobre a presença de pulsos distais ou a condição local do membro superior, há necessidade de exames clínicos repetidos e minuciosos após a redução urgente da luxação. Os autores referem o uso da oximetria de pulso, de fato uma ferramenta bastante disponível; no entanto, sua realização precisa ser esclarecida, ou seja, é preciso observar o formato da onda e não a oximetria complementada pela angiografia, de preferência a angiografia venosa, devido ao risco de trombose arterial. Na ausência de angiografia, a ultrassonografia com Doppler pode detectar a interrupção parcial ou total do fluxo arterial. Se houver a menor dúvida diante da persistência dos sinais de isquemia, a exploração cirúrgica é indicada. Este é o primeiro recurso nas luxações abertas do cotovelo.

As lesões da artéria braquial apresentam diversas nuances, desde simples contusão, espasmo, secção parcial a trombose (por retalho da íntima) e secção total. São bastante descritas em crianças com fraturas supracondilares, raramente em luxações abertas e ainda menos em luxações fechadas. 11

Essa lesão arterial pode estar associada ao dano neurológico por contiguidade das relações anatômicas neurovasculares e depende da direção da luxação e sua gravidade ( Tabela 1 ). 5 6 9 10 11 12 13

**Tabela 1:** Revisão de literatura da associação da lesão neurovascular na luxação de cotovelo em crianças

Autor	Número de casos (crianças)	Complicações	Resultados bons e excelentes (%)
Carlioz, Abols 5	58	Lesão do nervo ulnar – 2 casos	90%
Rassol 6	33	Lesão do nervo ulnar – 2 casos Lesão do nervo mediano – 1 caso Lesão do nervo radial – 1 caso **Lesão da artéria braquial – 1 caso**	67%
Subasi et al. 12	56	Lesão do nervo ulnar – 3 casos **Lesão do nervo mediano e lesão da artéria braquial – 1 caso**	48%
Louis et al. 13	1	**Ruptura da artéria braquial – 1 caso**	Não relatados
Hoffmann et al. 9	1	**Ruptura da artéria braquial – 1 caso**	Não relatados
Manouel et al. 10	1	**Ruptura da artéria braquial – 1 caso**	Não relatados
Brahmamdam et al. 11	1	**Ruptura da artéria braquial – 1 caso**	Não relatados

O reparo da secção da artéria braquial foi considerado em uma abordagem multidisciplinar com o cirurgião pediátrico, o cirurgião vascular e a equipe de anestesia e terapia intensiva.

O membro superior e o membro inferior foram preparados, este último para um possível enxerto venoso.

A avaliação da lesão revelou a ruptura total da artéria braquial na presença de um grande hematoma, que foi evacuado. Observou-se contusão do nervo mediano, sem perda de continuidade. Os nervos ulnar e radial estavam intactos, sem lesões tendinosas ou ligamentares óbvias.

Os nervos ulnar e radial estavam intactos, sem nenhum dano tendíneo ou ligamentar óbvio. Procedemos à redução da luxação posterior do cotovelo, que parecia estável após o procedimento.

A utilização de enxerto de veia safena reversa da coxa direita foi imprescindível devido à tensão da anastomose. Ao final da cirurgia, o paciente foi submetido à imobilização gessada.

A luxação do cotovelo associada à lesão da artéria braquial é uma emergência traumática muito rara na população pediátrica.

O exame clínico repetitivo é fundamental e deve ser complementado por ultrassonografia com Doppler arterial e/ou angiografia, sem retardar a exploração cirúrgica em caso de qualquer dúvida.
